# Susceptibility of Dental Caries Microcosm Biofilms to Photodynamic Therapy Mediated by Fotoenticine

**DOI:** 10.3390/pharmaceutics13111907

**Published:** 2021-11-10

**Authors:** Maíra Terra Garcia, Rafael Araújo da Costa Ward, Nathália Maria Ferreira Gonçalves, Lara Luise Castro Pedroso, José Vieira da Silva Neto, Juliana Ferreira Strixino, Juliana Campos Junqueira

**Affiliations:** 1 Department of Biosciences and Oral Diagnosis, Institute of Science and Technology/ICT, São Paulo State University/UNESP, São José dos Campos 12245-000, Brazil; maira.garcia@unesp.br (M.T.G.); r.ward@unesp.br (R.A.d.C.W.); nmf.goncalves@unesp.br (N.M.F.G.); lara.luise@unesp.br (L.L.C.P.); 2 Associate Laboratory of Sensors and Materials/LABAS, National Institute for Space Research, São José dos Campos 12227-010, Brazil; jose.v.silva@inpe.br; 3 Photobiology Applied to Health, Research and Development Institute IP&D, University of Vale do Paraiba/UNIVAP, São José dos Campos 12244-390, Brazil; juferreira@univap.br

**Keywords:** photodynamic therapy, dental caries, oral biofilms, Fotoenticine, chlorine, methylene blue

## Abstract

Photodynamic therapy (PDT) mediated by Fotoenticine^®^ (FTC), a new photosensitizer derived from chlorin e-6, has shown in vitro inhibitory activity against the cariogenic bacterium *Streptococcus mutans*. However, its antimicrobial effects must be investigated on biofilm models that represent the microbial complexity of caries. Thus, we evaluated the efficacy of FTC-mediated PDT on microcosm biofilms of dental caries. Decayed dentin samples were collected from different patients to form in vitro biofilms. Biofilms were treated with FTC associated with LED irradiation and analyzed by counting the colony forming units (log10 CFU) in selective and non-selective culture media. Furthermore, the biofilm structure and acid production by microorganisms were analyzed using microscopic and spectrophotometric analysis, respectively. The biofilms from different patients showed variations in microbial composition, being formed by streptococci, lactobacilli and yeasts. Altogether, PDT decreased up to 3.7 log10 CFU of total microorganisms, 2.8 log10 CFU of streptococci, 3.2 log10 CFU of lactobacilli and 3.2 log10 CFU of yeasts, and reached eradication of *mutans* streptococci. PDT was also capable of disaggregating the biofilms and reducing acid concentration in 1.1 to 1.9 mmol lactate/L. It was concluded that FTC was effective in PDT against the heterogeneous biofilms of dental caries.

## 1. Introduction

Dental caries is a multifactorial biofilm-mediated disease characterized by the mineral loss of tooth hard tissues [1,2]. Caries development is a result of dental biofilm acidification from carbohydrate consumption that alters the oral ecology, favoring the growth of acidogenic and acid-tolerating species [2,3,4]. Despite the restorative treatments available for dental caries, dentists commonly encounter failures in restorations associated with secondary caries [5]. Therefore, a proper disinfection of the dental cavity before the restorative procedure is essential to avoid the growth of bacteria under restorative material and caries recurrence [6,7]. In this context, alternative adjuvant methods to restorative treatment have been extensively investigated, such as antimicrobial photodynamic therapy (PDT).

The antimicrobial effect of PDT is based on a photophysical reaction through the association of a photosensitizer, light at an appropriate wavelength (usually from the visible to near infrared spectrum) and molecular oxygen [8,9,10,11,12]. In this process, the photosensitizer is activated by light to produce reactive oxygen species (ROS) that kill the microbial cells via an oxidative burst [8,13]. Therefore, the efficacy of PDT depends on the type of photosensitizer, parameters of irradiation, and application site [14]. In the last decades, different photosensitizers have been investigated, including phenothiazines and chlorine derivatives [8,9,15,16]. Phenothiazines, such as methylene blue (MB), are widely studied and known, being already approved for clinical dental application in several countries around the world [17]. Chlorines are tetrapyrrolic substances derived from chlorophyll that have gained attention due to their photodynamic effects on a large number of pathogens [18]. Some derivatives of chlorines have been described as potential photosensitizers with high absorption in the red region of the visible spectrum and mechanism of action predominantly via type II reaction, producing singlet oxygen [19]. Among them, Fotoenticine (FTC), a new photosensitizer derived from chlorin e-6, has demonstrated strong photodynamic activity against the cariogenic bacterium *Streptococcus mutans* [9,19,20].

Recently, Terra-Garcia et al. [9] and Nie et al. [19] verified that PDT mediated by FTC was able to reduce the viability of biofilms formed by a reference *S. mutans* strain (UA159). In both studies, the antimicrobial effects of PDT with FTC were greater than MB, suggesting that FTC can be a useful photosensitizer in the control of *S. mutans*. Later, our research group reported that photodynamic inactivation by FTC was extended to several clinical strains of *S. mutans* isolated from patients with the active disease [20]. In all of these clinical strains, FTC-mediated PDT was able to eliminate the viable cells and disrupt the dense structure of *S. mutans* biofilms [20].

Although previous studies have suggested that PDT mediated by FTC can be a promising strategy to prevent or treat caries, these studies were limited to monospecies biofilms that do not represent the microbial complexity of dental biofilms. It is known that dental biofilms are composed of different microbial species that form a highly complex and organized structure on the tooth tissues, in which each microorganism has specific positions and functions. More than 700 bacterial species have been identified from dental biofilm, and approximately 40 species were already associated with caries [21]. In addition to *S. mutans*, other acidogenic and aciduric microorganisms in the biofilm may play an important role in dental caries, such as *Streptococcus sobrinus*, *Lactobacillus* spp., *Veillonella* spp., *Actinomyces* spp. and *Candida* spp. [22].

To overcome the limitations of in vitro biofilm studies with one or three selected species, many researchers have introduced different models of microcosm biofilms. Microcosms are more reliable models to simulate the in situ conditions of the oral cavity. Microcosm biofilms consist of a set of microorganisms from the natural oral microbiota that are formed in vitro using samples collected from the oral cavity, such as saliva or biofilms [23]. Thus, microcosm biofilms are capable of reproducing the biodiversity, ecological interactions, acid production and pH conditions of dental biofilms in the human oral cavity [24]. Using a microcosm model, the objective of this study was to evaluate the antimicrobial activity of PDT mediated by FTC on dental caries biofilm, investigating its effects on the microbial cells, biofilm structure and acid production.

## 2. Materials and Methods

### 2.1. Collection of Dental Caries Samples

This study was approved by the Ethics Committee for Research in Human Beings of the Institute of Science and Technology (ICT/UNESP) under protocol number 158617/2019, date of approval: 13 November 2020). Three patients of the dental clinics in the city of São José dos Campos were selected. Inclusion criteria were age between 20 and 40 years and presence of at least one dentin caries lesion in a molar tooth. The non-inclusion criteria were the use of antibiotics in the last 90 days and presence of caries lesions with pulp exposure.

The collection of samples from infected dentin was carried out after anesthesia and absolute isolation with a rubber dam. The material was collected with a sterile curette, transferred to brain heart infusion (BHI) broth with 20% glycerol and stored in a freezer at −80 °C. Teeth were then restored according to pre-determined treatment for each patient.

### 2.2. Preparation of Photosensitizer and Light Source

**Figure 1:**
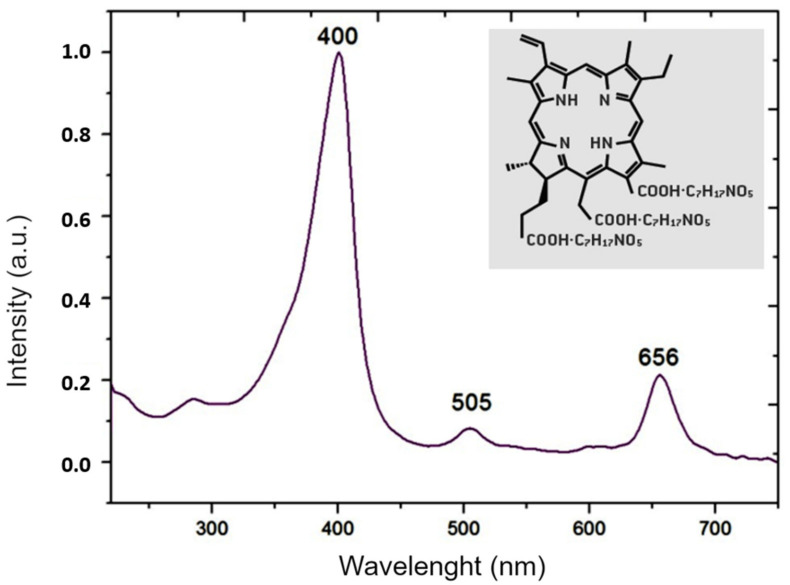
Chemical structure [25] and absorption spectrum of Fotoenticine (200 μg/mL in 0.9% NaCl) determined with a spectrophotometer (DS-11, Denovix, DE, USA).

The photosensitizer Fotoenticine^®^ (Nuevas Tecnologias Cientificas, NTC, Lianera Asturias, Spain) obtained at a concentration of 6.89 mg/mL was sterilized by filtration on membranes with pores of 0.22 µm (MFS, Dublin, Ireland) and stored in dark conditions. The chemical structure and absorption spectrum of the Fotoenticine^®^ are showed in Figure 1. The light source was a LED at 660 nm (IrradLED^®^, Biopdi, São Carlos, Brazil) with a power density of 42.8 mW/cm^2^, energy density of 30 J/cm^2^ and exposure time of 700 s.

### 2.3. Preparation of Bovine Tooth Specimens

**Figure 2:**
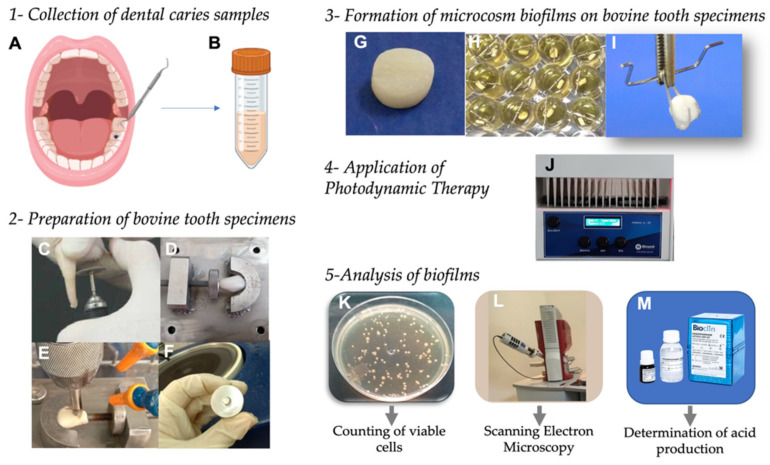
(**A**) Representation of sample collection from infected dentin of patient. (**B**) Sample transferred to brain heart infusion (BHI) broth with 20% glycerol. (**C**) Separation of crown from the root of bovine tooth using a straight handpiece with a diamond disc. (**D**) Crown attached to a circular sample cutter with the buccal side facing upwards. (**E**) Tooth cut with a trephine drill. (**F**) Specimen inserted into a metal matrix for polishing. (**G**) Bovine specimen prepared. (**H**) Specimen positioned in the metal wire and submerged in BHI broth to form the biofilms. (**I**) Biofilm formed on bovine specimen. (**J**) Irradiation of biofilms with LED at 660 nm. (**K**) Colonies formed on BHI agar. (**L**) Scanning electron microscope. (**M**) Lactic dehydrogenase LDH UV K014 (Bioclin) to determine the acid production.

Bovine teeth were employed as substrate to form in vitro biofilms following the methodology described by Garcia et al. [9] with some modifications. Mandibular incisor teeth were used to prepare specimens measuring 4 mm in diameter and 2 mm in thickness. In summary, the crowns were separated from the roots using a straight handpiece with a diamond disc. The crowns were attached to a circular sample cutter with the buccal side facing upwards and cut with a trephine drill. Then, specimens were inserted into a metal matrix (with the dentin surface positioned in its interior) and polished with sandpaper discs of decreasing grain size in a low-speed water-cooling system (DP-10, Panambra Industrial e Técnica, São Paulo, Brazil) (Figure 2). The final thickness was confirmed with a digital caliper (Starrett, São Paulo, Brazil). Finally, the specimens were stored in a 0.1% (*v*/*v*) thymol solution at 4 °C.

### 2.4. Formation of Microcosm Biofilms on Bovine Tooth Specimens

Microcosm biofilms were formed according to the methodology of Méndez et al. with some modifications [24]. Dental caries samples stored in freezer were thawed and homogenized. Then, 400 μL were transferred to 20 mL of BHI broth supplemented with 5% sucrose and incubated at 37 °C in 5% CO_2_ for 48 h. The growth was centrifuged and washed twice in phosphate buffered saline (PBS). The pellet was resuspended in 20 mL of PBS. An inoculum of 225 μL of this microbial suspension was added in each well of 24-well microplates, containing the bovine tooth specimen suspended by a metal wire and submerged in 2 mL of BHI broth with 5% sucrose. The plates were incubated at 37 °C for 120 h to form the biofilms, under 5% CO_2_ pressure since most cariogenic bacteria are capnophilic [26]. Every 24 h, the wells were washed (2×) with PBS, and 2 mL of a fresh BHI broth with 5% sucrose was replaced.

### 2.5. Application of Photodynamic Therapy on Microcosm Biofilms

After biofilm formation, the specimens were removed from the metal wire support, transferred to a new well and submerged in 200 μL of photosensitizer or PBS. To obtain an antimicrobial effect on biofilms, the photosensitizer was used at a concentration of 0.6 mg/mL that is considered able to diffuse through *S. mutans* biofilms [20]. The plates were shaken for 15 min (pre-irradiation time) in dark conditions. After that, the plates were taken to the IrradLED^®^ and irradiated according to the parameters described above. The control groups not irradiated remained in dark conditions for the same period. According to these procedures, four groups of microcosm biofilms were established: treated with FTC and irradiated (FTC + L+), treated with PBS and irradiated (P − L+), treated with FTC in the dark (FTC + L−) and treated with PBS in the dark (P − L−).

### 2.6. Analysis of Biofilms by Counting the Number of Viable Cells

The specimens containing the treated biofilms (five per group) were transferred to Falcon tubes containing 4 mL of PBS. The adhered biofilms were detached using an ultrasonic homogenizer (Sonopuls HD2200, Bandelin Eletronic, Berlin, Germany) with a power of 7 W for 30 s. Serial dilutions of the biofilm suspension were performed and plated on BHI agar for counting the total number of microorganisms, as well as on the following selective culture media: Mitis Salivarius for counting streptococci, Mitis Salivarius Bacitracin Sucrose (MSBS) agar for mutans streptococci, Rogosa agar for lactobacilli, and Sabouraud Dextrose agar with chloramphenicol for yeasts. Next, the plates were incubated at 37 °C for 48 h to determine the number of colony forming units (CFU).

### 2.7. Analysis of Biofilms by Scanning Electron Microscopy (SEM)

After the treatments, the specimens with biofilms (two per group) were submerged in 1 mL of 2.5% glutaraldehyde (1 h) for cell fixation. After that, they were dehydrated with 1 mL of increasing ethanol concentrations (10, 25, 50, 75 and 90%), remaining for 20 min at each concentration. Finally, the biofilms were added to absolute ethanol for 1 h. The dehydrated biofilms were incubated at 37 °C for 24 h, and then placed in aluminum stubs and covered with gold for 160 s at 40 mA (Denton Vacuum Desk II, Denton Vacuum, Moorestown, NJ, USA). After metallization, the biofilms were analyzed in the scanning electron microscope (JEOL JSM-7900, JEOL, Peabody, MA, USA).

### 2.8. Determination of Lactic Acid Production by the Microbial Cells in Biofilms

The specimens with the treated biofilms (5 per group) were transferred to Falcon tubes containing 5 mL of PBS and incubated at 37 °C for 3 h. Then, 1 mL of each tube was placed in a freezer at −80 °C for 5 min to stop the acid production. To verify the concentrations of lactate, the enzymatic method of lactic dehydrogenase was used according to the manufacturer’s instructions (Lactic dehydrogenase LDH UV K014, Bioclin, Belo Horizonte, Brazil). Absorbance was measured at 340 nm using a microplate spectrophotometer (Epoch, Biotek, Winooski, VT, USA), and the obtained values were expressed as mmol lactate/L.

### 2.9. Statistical Analysis

The results obtained in the viable cell counts (log10 CFU) and acid production were analyzed by ANOVA and Tukey test using the GraphPad Prism 8.4.3 program (GraphPad Software, Inc., San Diego, CA, USA). For statistical analysis, five biofilms per group were used. The assays were repeated twice. Level of significance of 5% was adopted in all the analyses.

## 3. Results

### 3.1. Effects of FTC-Mediated PDT on Viable Cells of Microcosm Biofilms

**Figure 3:**
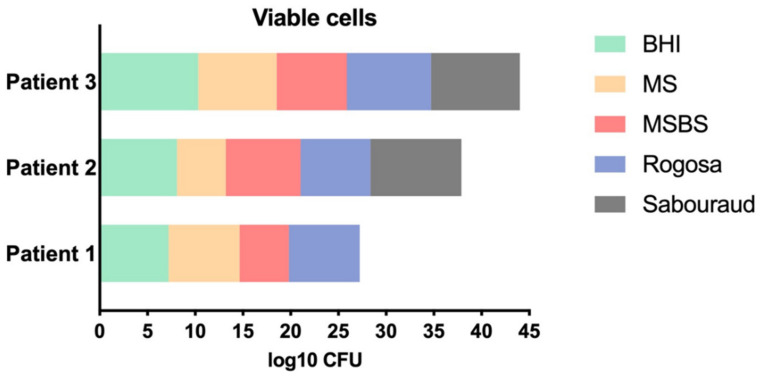
Viable cell counts (log10 CFU) of microcosm biofilms from patients 1, 2 and 3 according to the following culture mediums: brain heart infusion (total number of microorganisms), Mitis Salivarius (streptococci), Mitis Salivarius Bacitracin Sucrose (mutans streptococci), Rogosa (lactobacilli), and Sabouraud Dextrose with chloramphenicol (yeasts).

In the viable cell counts of the non-treated biofilms (control group P − L−), the microbial growth in non-selective culture media (BHI) was 7.21 log10 CFU for patient 1, 8.09 log10 CFU for patient 2 and 10.31 log10 CFU for patient 3. These data showed that the microcosm biofilm of patient 3 had the highest quantity of microorganisms, followed by patient 2 and 1. In the selective culture media, all patients showed cariogenic biofilms formed by streptococci (5 to 8 log10 CFU), mutans streptococci (5 to 7 log10 CFU) and lactobacilli (7 to 8 log10 CFU). However, the presence of yeasts was found only in microcosm biofilms of patients 2 and 3 (9 log10 CFU) (Figure 3).

**Figure 4:**
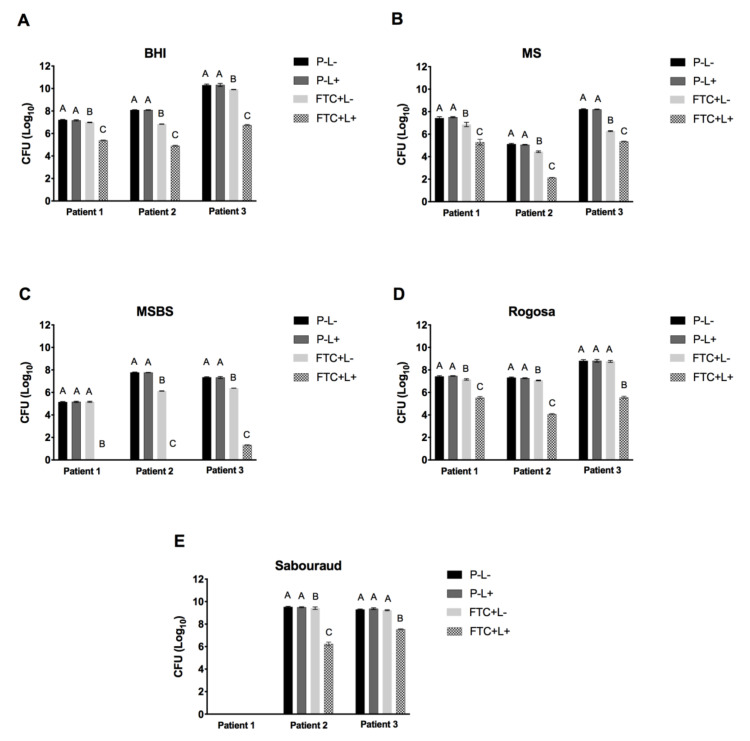
Means of log10 CFU and SD of microcosm biofilms (patients 1, 2 and 3) obtained in the following groups: treated with PBS in the dark (P − L−), treated with PBS and irradiated (P − L+), treated with FTC in the dark (FTC + L−) and treated with FTC and irradiated (FTC + L+). (**A**) Total microorganisms in brain heart infusion agar. (**B**) Streptococci in Mitis Salivarius agar. (**C**) Mutans streptococci in Mitis Salivarius Bacitracin Sucrose (MSBS) agar. (**D**) Lactobacilli in Rogosa agar. (**E**) Yeasts in Sabouraud Dextrose agar with chloramphenicol.

In relation to the viable cell counts of the biofilms treated with PDT (FTC + L+), we observed statistically significant microbial reductions relative to the groups P − L− and P − L+. These microbial reductions occurred in both selective and non−selective culture media for all the patients studied. In the non-selective medium, the FTC + L+ caused reductions from 1.8 to 3.7 log10 CFU for the total microorganisms compared to the P − L− group. Significant microbial reductions were also found in the FTC + L+ group in all the selective culture media, indicating that the FTC-mediated PDT was effective in inhibiting the growth of different microorganisms associated with cariogenic biofilms, including streptococci, mutans streptococci, lactobacilli and yeasts. In addition, the group treated with FTC alone (FTC + L−) showed a reduction from 0.2 to 1.2 log10 CFU in comparison to the P − L− group, suggesting a slightly toxic effect of FTC in dark conditions (Figure 4).

**Figure 5:**
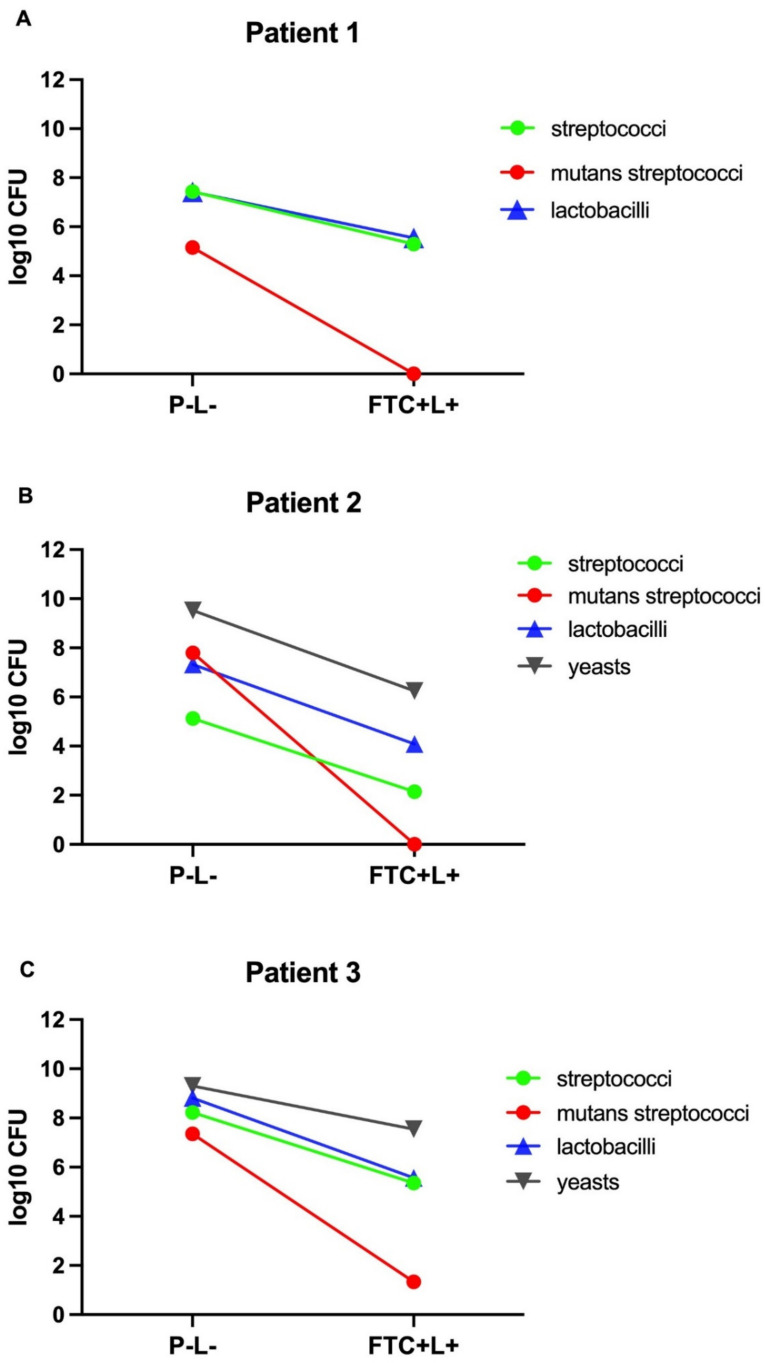
Microbial reductions of streptococci, mutans streptococci, lactobacilli and yeasts expressed in log10 CFU obtained in the group treated with FTC and irradiated (FTC + L+) in comparison to the control group treated with PBS in the dark (P − L−). (**A**) Microcosm biofilm from patient 1. (**B**) Microcosm biofilm from patient 2. (**C**) Microcosm biofilm from patient 3.

Considering the biofilms of all patients, the mean of microbial reductions achieved by FTC-mediated PDT (in comparison to the P − L− group) ranged from 2.1 to 2.8 log10 CFU for streptococci, 1.8 to 3.2 log10 CFU for lactobacilli and 1.7 to 3.2 log10 CFU for yeasts. Interestingly, mutans streptococci had a greater susceptibility to PDT relative to the other microorganisms. FTC-mediated PDT caused a mutans streptococci reduction of 6.02 log10 CFU for the biofilm of patient 3 and a total eradication for patients 1 and 2 (Figure 5).

### 3.2. Effects of FTC-Mediated PDT on Microcosm Biofilm Structures

The SEM images confirmed that all the caries samples collected from patients were able to provide an in vitro formation of microcosm biofilms on the surface of bovine dentin specimens. However, the morphology and structure of the biofilms varied according to the experimental groups. In all the patient samples, the P − L− and P − L+ groups presented a dense and compact biofilm, forming a complex three-dimensional structure. These biofilms were composed of a large quantity of microbial aggregates embedded in an extracellular matrix, covering the entire dentin surface. Different morphologies of microorganisms were found, including coccus, coccobacilli, filamentous bacilli, fusiform bacilli and fungal hyphae, which represented the microbial diversity of cariogenic biofilms.

**Figure 6:**
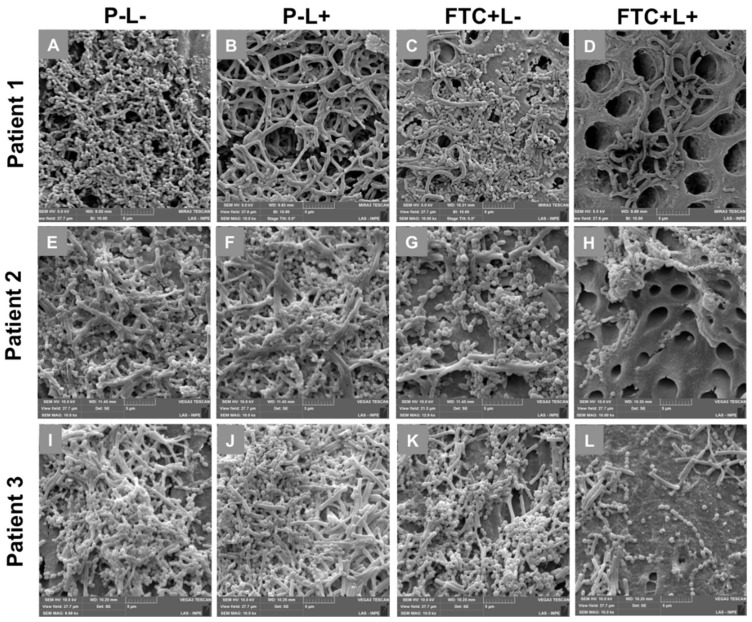
Scanning electron microscopy images according to the groups: treated with PBS in the dark (P − L−), treated with PBS and irradiated (P − L+), treated with FTC in the dark (FTC + L−) and treated with FTC and irradiated (FTC + L+). (**A**–**D**) Microcosm biofilm from patient 1. (**E**–**H**) Microcosm biofilm from patient 2. (**I**–**L**) Microcosm biofilm from patient 3.

The FTC + L− group also showed a dense biofilm formed by a large number of microorganisms; however, the biofilm density was lower than that in the P − L− and P − L+ groups. When the microcosm biofilms were treated with PDT (FTC + L+ group), a substantial reduction in the amount of microorganisms adhered to tooth surface was clearly observed. The PDT was capable of disaggregating the biofilms, exposing the dentin surface formed by several dentinal tubules. The microorganisms on dentin surface exhibited few cellular aggregates or isolated cells, allowing a more precise observation of microbial morphologies (Figure 6).

### 3.3. Influence of FTC-Mediated PDT on Acid Production by the Microbial Cells of Microcosm Biofilms

In the biofilms not treated with PDT (P − L−, P − L+ and FTC + L− groups), the results of acid production ranged from 1.95 to 1.96 mmol lactate/L for patient 1, from 2.79 to 2.82 mmol lactate/L for patient 2, and from 3.80 to 3.84 mmol lactate/L for patient 3. As with the results obtained in the viable cell counts, patient 3 showed the highest quantity of acid production, followed by patient 2 and 1. These results indicated that the acidogenic capacity of caries microcosm biofilms was related to the number of microbial cells.

**Figure 7:**
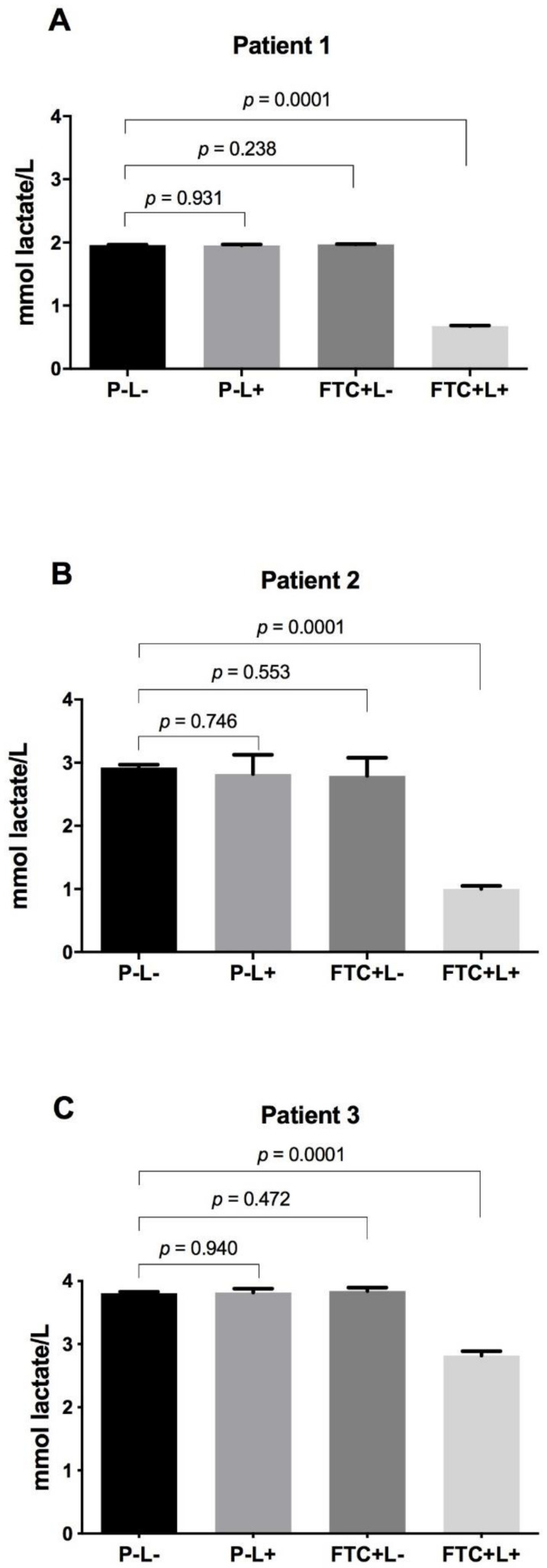
Means and SD of lactic acid concentration (mmol lactate/L) for the groups: treated with PBS in the dark (P − L−), treated with PBS and irradiated (P − L+), treated with FTC in the dark (FTC + L−) and treated with FTC and irradiated (FTC + L+). (**A**) Microcosm biofilm from patient 1. (**B**) Microcosm biofilm from patient 2. (**C**) Microcosm biofilm from patient 3.

The biofilms treated with PDT (FTC + L+ group) showed statistically significant reductions of acid production when compared to the P − L− group for all patients studied. The acid reductions caused by PDT were 1.28, 1.92 and 0.99 mmol lactate/L, respectively, for patients 1, 2 and 3 (Figure 7). In general, it was possible to observe that PDT mediated by FTC was able to kill the microbial cells with consequent disruption of biofilm structure and decreased acidogenicity.

## 4. Discussion

Cariogenic biofilms are active and complex ecosystems rich in organic acid [27], which form a spatial and ordered organization of microbial communities on tooth surfaces [28]. The dental surfaces and the development of caries lesions provide different microenvironments that allow the colonization of adapted microbial communities, leading to variations in the bacterial amount and microbial composition of cariogenic biofilms according to the caries stage [29,30]. In addition, several individual factors can influence the biofilm diversity, such as the host’s diet, oral hygiene habits, tooth morphology, saliva composition, and discrepancies in the immune system [27,31]. Considering the variations in cariogenic biofilms among different individuals, in this study we collected samples from three patients with dentin caries to form in vitro microcosm biofilms and to investigate the potential of FTC-mediated PDT for the control of dental caries.

The microcosm biofilm obtained from patient 3 (10.31 log10 CFU) showed the highest total microbial count, followed by patient 2 (8.09 log10 CFU) and 1 (7.21 log10 CFU). In the three patients analyzed, the results were superior to those found by Mendéz et al. [24], who obtained a total microbial count of approximately 6.5 log10 CFU in the microcosm biofilms from dentin caries of children. Possibly, these differences can be attributed to the age of patients because in our study, only adults were included. However, Rudney et al. [32] did not observe differences in the quantity of microorganisms between adult and pediatric patients when microcosm biofilms were formed from saliva or dental plaque. The total microbial count ranged from 8 to 10 log10 CFU, independently of the inoculum source. Although the quantity of microorganisms formed in microcosm biofilms can vary depending on the study in the literature, it is evident that the microcosm model is able to provide very dense biofilms that can be useful for a large number of in vitro studies.

In this study, the microcosm biofilms from all patients were composed of streptococci, mutans streptococci and lactobacilli. In addition, the microcosm biofilms of patients 2 and 3 presented yeasts as well. Indeed, caries is closely associated with high counts of streptococci (particularly *Streptococcus mutans*), lactobacilli and yeasts of the genus *Candida* [2,33]. *Streptococcus* spp. and *Lactobacillus* spp. exhibit special properties that make them cariogenic bacteria, including the ability to adhere to dental surfaces, to produce a high quantity of acids from fermentable sugars, and to survive in an acid environment [34]. *Candida* species have been frequently isolated from dental caries lesions and also display acidogenic and aciduric properties. The presence of *Candida albicans* in cariogenic biofilms seems to enhance the virulence of *S. mutans* and, consequently, the severity of dental caries [35,36]. Many other bacterial genera have also been associated with cariogenic biofilms, such as *Actionomyces*, *Bifidobacteria*, *Veilonella*, *Prevotella* [2,34], and more recently, *Scardovia* [2]. However, the present study was limited to the identification of streptococci, mutans streptococci, lactobacilli and yeasts in the microcosm biofilms. Therefore, additional studies with a focus on other microbial groups are required, especially those that use molecular tools.

The viable cell counts in the microcosm biofilms of patients ranged from 5 to 8 log10 CFU for streptococci, 5 to 7 log10 CFU for mutans streptococci, 7 to 8 log10 CFU for lactobacilli and 0 to 9 log10 CFU for yeasts. Similar results were found by Vertuan et al. [37], Câmara et al. [38], and Mendéz et al. [24], who obtained bacterial counts between 6 to 7 log10 CFU for streptococci, 5 to 7 log10 CFU for mutans streptococci and 6 to 7 log10 CFU for lactobacilli in microcosm biofilms from oral samples. Nonetheless, these authors did not investigate the presence of yeasts in the microcosm biofilms formed in their studies.

When the FTC-mediated PDT was applied on microcosm biofilms, the viable cell numbers of all microbial groups studied were significantly decreased. In general, the microcosm biofilms from patients showed reductions between 2.1 to 2.8 log10 CFU for streptococci, 6.0 log10 CFU to total inhibition for mutans streptococci, 1.8 to 3.2 log10 CFU for lactobacilli and 1.7 to 3.2 log10 CFU for yeasts after the PDT treatment. Interestingly, the antimicrobial effects of FTC observed in our study were more pronounced than other photosensitizers cited in the literature. Using microcosm biofilms from dentin caries of patients, some authors found microbial reductions of 1.5, 1.3 and 1.9 log10 CFU with the photosensitizer methylene blue [24] and 1.5, 1.7 and 2.3 log10 CFU when curcumin was used as photosensitizer [39]. Probably, the greater photodynamic activity of FTC can be related to the positive charges of chlorins, action predominantly via type II mechanism with singlet oxygen quantum yields from 0.5 to 0.8 [8], and high capacity to penetrate into the bacterial cells [13,19].

To our knowledge, this is the first study to investigate the effects of FTC-mediated PDT on microcosm biofilms. Until now, this new photosensitizer has been studied in single biofilms formed by *S. mutans*. Terra-Garcia [20] applied the FTC-mediated PDT on single biofilms of *S. mutans* formed by the reference strain UA159 or clinical strains isolated from dental caries, verifying microbial reductions between 5 to 6 log10 CFU for the clinical strains and total inhibition for the reference strain. Using a similar photosensitizer also derived from chlorin-e6, Nie et al. [19] found a bacterial reduction of 5 to 6 log10 CFU of *S. mutans* (UA159) in single biofilms treated with PDT. Promisingly, the results of the present study using microcosm models showed that *S. mutans* remained susceptible to PDT mediated by FTC even when mixed in a complex polymicrobial biofilm.

In addition to remaining susceptible to FTC-mediated PDT in microcosm biofilms, mutans streptococci were the microorganisms most susceptible to this therapy, being totally eradicated in the microcosm biofilms from patients 1 and 2. In relation to the biofilm of patient 3, mutans streptococci were also more susceptible to PDT than lactobacilli and yeasts. We hypothesized that the highest susceptibility of *S. mutans* to PDT mediated by FTC could be associated with the growth rate, microbial cell composition, or spatial organization of biofilms. The growth rates and generation times of *S. mutans* cells were more elevated than those of *Lactobacillus* cells [40], which could influence the structure and permeability of cell membrane. The cell composition of bacteria and yeast is also considered an important factor in the susceptibility to PDT. Due to the presence of a cell wall with porous layer of peptidoglycan, Gram-positive bacteria such as *S. mutans* are more susceptible to PDT than fungal cells such as *Candida* spp. that present a cell wall of chitin with a less porous layer of beta-glucan [41]. In addition, the spatial organization of *S. mutans* cells with other microorganisms in polymicrobial biofilms can affect their susceptibility to antimicrobial treatments [42]. Using dual-species model biofilms, previous studies showed that *S. mutans* use *Candida* cells as support for their adherence [43,44]. According to these authors, *S. mutans* cells have affinity for the *C. albicans* hyphae, appearing positioned over the hyphal surface in SEM images [43,44]. This fact may leave *S. mutans* more exposed to PDT action. However, future studies are required to unveil the spatial structure of cariogenic biofilms and to develop therapies targeting the biogeography of polymicrobial infections [28].

The SEM analysis performed in the present study confirmed the capacity of microcosm biofilms in reproducing the dental caries microbiome. The biofilms of non-treated groups showed a spatial structure formed by heterogeneous and complex communities embedded in an extracellular matrix. The FTC-mediated PDT was able to decrease the number of microbial cells and to disaggregate the biofilm structure, exposing the dentin surface. In a review article, Hu et al. [13] reported that PDT not only kills the bacterial and fungal cells present in biofilms, but the ROS generated can concomitantly degrade the matrix structure by attacking several biomolecules. During the PDT reaction, the burst of ROS causes oxidative damage in multiple non-specific targets, such as amino acids, nucleic acid bases, lipids, etc. Therefore, PDT can disaggregate the biofilms by a synergistic action on microbial cells and extracellular matrix. These factors make PDT an attractive approach for chronic biofilm infections [13].

Since the production of organic acids by microbial cells in biofilms is a determinant of the development of dental caries [34], in this study we also evaluated the influence of PDT on the acidogenicity of biofilms. The PDT mediated by FTC caused a substantial reduction in lactic acid production for the three biofilms analyzed in this study, probably as a consequence of the decrease in the number of acidogenic bacteria. On the other hand, Mendez et al. [24,39] did not observe reductions in lactic acid production when the dentin microcosm biofilms were treated with PDT mediated by methylene blue [24] or curcumin [39]. These authors did not find correlations between the reductions in CFU counts and lactic acid production, raising the hypothesis that the surviving cells could have increased their acid production capacity, which resulted in maintaining the acidogenicity of the biofilms. However, we think that the bacterial reductions caused by PDT with MB [24] or curcumin [39] may not have been enough to result in a decrease in acid production because the CFU reductions were lower than the microbial reductions found in our study using FTC.

In summary, we concluded that PDT mediated by FTC, a photosensitizer derived from chlorin e-6, has antimicrobial activity against dental caries microcosm biofilms. FTC-mediated PDT led to significant reductions in total microorganisms, streptococci, mutans group streptococci, lactobacilli and yeasts. Mutans group streptococci were the microorganisms most susceptible to PDT, showing total eradication after this therapy. In addition, PDT with FTC was able to disaggregate the biofilm structure and to reduce the lactic acid concentration. Promisingly, FTC can be an attractive photosensitizer for PDT targeted to the control of cariogenic biofilms and dental caries.
